# Fully Oxygen-Tolerant Visible-Light-Induced ATRP of Acrylates in Water: Toward Synthesis of Protein-Polymer Hybrids

**DOI:** 10.1021/acs.macromol.2c02537

**Published:** 2023-02-20

**Authors:** Kriti Kapil, Arman Moini Jazani, Grzegorz Szczepaniak, Hironobu Murata, Mateusz Olszewski, Krzysztof Matyjaszewski

**Affiliations:** Department of Chemistry, Carnegie Mellon University, 4400 Fifth Avenue, Pittsburgh, Pennsylvania 15213, United States

## Abstract

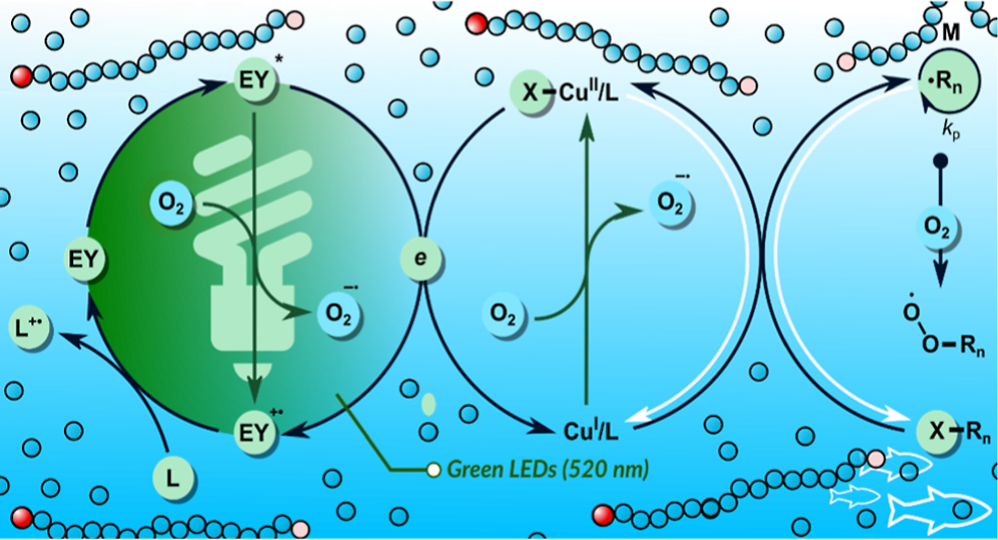

Over the last decade, photoinduced ATRP techniques have been developed to harness the energy of light to generate radicals. Most of these methods require the use of UV light to initiate polymerization. However, UV light has several disadvantages: it can degrade proteins, damage DNA, cause undesirable side reactions, and has low penetration depth in reaction media. Recently, we demonstrated green-light-induced ATRP with dual catalysis, where eosin Y (EYH_2_) was used as an organic photoredox catalyst in conjunction with a copper complex. This dual catalysis proved to be highly efficient, allowing rapid and well-controlled aqueous polymerization of oligo(ethylene oxide) methyl ether methacrylate without the need for deoxygenation. Herein, we expanded this system to synthesize polyacrylates under biologically relevant conditions using Cu^II^/Me_6_TREN (Me_6_TREN = tris[2-(dimethylamino)ethyl]amine) and EYH_2_ at ppm levels. Water-soluble oligo(ethylene oxide) methyl ether acrylate (average *M*_n_ = 480, OEOA_480_) was polymerized in open reaction vessels under green light irradiation (520 nm). Despite continuous oxygen diffusion, high monomer conversions were achieved within 40 min, yielding polymers with narrow molecular weight distributions (1.17 ≤ *D̵* ≤ 1.23) for a wide targeted DP range (50–800). In situ chain extension and block copolymerization confirmed the preserved chain end functionality. In addition, polymerization was triggered/halted by turning on/off a green light, showing temporal control. The optimized conditions also enabled controlled polymerization of various hydrophilic acrylate monomers, such as 2-hydroxyethyl acrylate, 2-(methylsulfinyl)ethyl acrylate), and zwitterionic carboxy betaine acrylate. Notably, the method allowed the synthesis of well-defined acrylate-based protein-polymer hybrids using a straightforward reaction setup without rigorous deoxygenation.

## Introduction

Reversible deactivation radical polymerization (RDRP) techniques,^1−3^ including atom transfer radical polymerization (ATRP),^4−7^ reversible addition–fragmentation chain-transfer (RAFT) polymerization,^8−10^ and nitroxide-mediated polymerization (NMP),^11^ enable facile synthesis of tailor-made polymers with a predetermined molecular weight, low dispersity, and unique macromolecular architecture. In these methods, an equilibrium is established between dormant and active species, in which dormant forms predominate. As a result, the fraction of terminated chains is diminished.

ATRP is a reversible redox process, typically catalyzed by Cu complexes,^12,13^ in which a halogen atom (X) is transferred from the dormant C(sp^3^)–X polymer chain end to the Cu^I^/L activator, forming a propagating radical and X–Cu^II^/L deactivator.^14,15^ However, oxygen interferes with initiating and propagating radicals by formation of peroxy radicals, as in any other radical polymerization.^16^ Furthermore, molecular oxygen can oxidize the Cu^I^/L activator to its inactive form (Cu^II^/L), inhibiting polymerization.^17^ For this reason, normal ATRP is carried out under strictly anaerobic conditions after a rigorous deoxygenation process by purging with inert gas or multiple freeze-pump-thaw cycles.^18^ As a result, conventional ATRP techniques could be challenging for non-experts. In addition, the synthesis of polymer biohybrids by ATRP can also pose difficulties because proteins or nucleic acids are susceptible to mechanical degradation during deoxygenation.^19,20^ Therefore, the development of oxygen-tolerant ATRP techniques is important.^21−33^

Over the last two decades, several ATRP techniques based on regenerating the activator have been developed.^4,26,34−39^ When the Cu^II^/L is continuously converted back to Cu^I^/L, the catalytic system can act as an oxygen scavenger and provide oxygen tolerance.^40^ The Cu^I^/L activator can be regenerated by reducing agents,^41−44^ electro-,^45,46^ photo-,^47−49^ or mechanochemical stimuli.^39,50,51^ Among these methods, photoinduced ATRP (photo-ATRP) has attracted the most interest because of its mild reaction conditions and ability to temporally and spatially control polymerization.^47,48,52,53^

**Figure 1:**
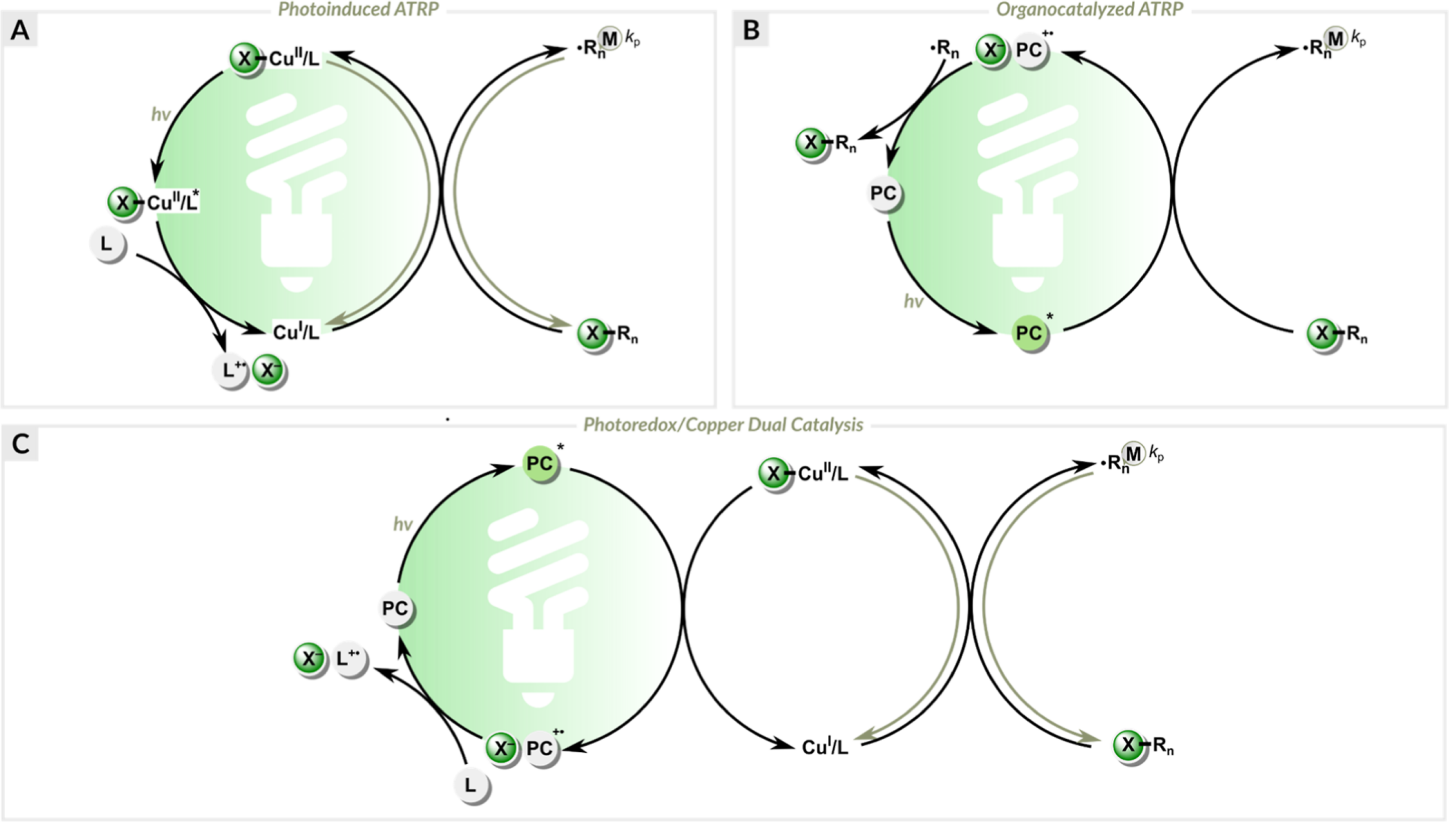
Different modes of radical generation in photoinduced ATRP. (a) Conventional photo-ATRP. (b) Organocatalyzed ATRP. (c) ATRP with photoredox/copper dual catalysis.

There are three modes to generate radicals in photoinduced ATRP. In conventional photo-ATRP, the Cu^II^/L in the excited state is reduced by an amine-based ligand, leading to the formation of Cu^I^/L and an amine radical cation (Figure 1a).^38^ Then, the generated activator Cu^I^/L reacts with a C(sp^3^)–X polymer chain end to form a carbon radical and the X–Cu^II^/L deactivator. Oxygen tolerance in this approach can be achieved using an excess ligand.^54−57^ However, photoinduced Cu-catalyzed ATRP typically requires the use of biocidal UV light.^26,58,59^ In organocatalyzed ATRP (O-ATRP), the dormant polymer chain is directly activated by electron transfer from a photoredox catalyst (PC) in the excited state (Figure 1b).^60−64^ O-ATRP is compatible with a wide range of visible light but is mainly limited to methacrylates and organic solvents.^65−71^ ATRP with dual catalysis uses copper complexes to attain a controlled radical propagation and PCs to trigger and drive polymerization (Figure 1c).^24,72−80^ The photoredox/copper dual catalysis overcomes the challenges of using biocidal UV light, poor oxygen tolerance, and limited monomer scope.

Water is the ideal medium for modifying biomolecules by ATRP.^19,81^ However, the high equilibrium constant of ATRP in water leads to a high concentration of radicals, which may result in many dead chains.^82,83^ There is also a significant dissociation of the [X–Cu^II^/L]^+^ deactivator to a free halide anion and the “naked” [Cu^II^/L]^2+^, which cannot deactivate propagating radicals.^84,85^ In addition, some ligands can cause disproportionation of Cu^I^ species to Cu^II^ and Cu(0).^86^ Also, hydrolysis of the C(sp^3^)–X bond can cause a significant problem in aqueous media, leading to loss of chain-end functionality since the C(sp^3^)–OH cannot participate in further chain growth. The rate of hydrolysis of the C(sp^3^)–X bond depends on the type of halogen (alkyl bromides are more easily hydrolyzed than chlorides) and on the substitution of the C(sp^3^) atom (secondary halides are more prone to hydrolysis than tertiary).^87−89^ For this reason, the vast majority of developed methods enable well-controlled polymerization of methacrylates, while ATRP of acrylates in water is still considered a challenge.^87^ Only a few successful examples of aqueous photo-ATRP of hydrophilic acrylates have been reported.^90−93^

**Figure 2:**
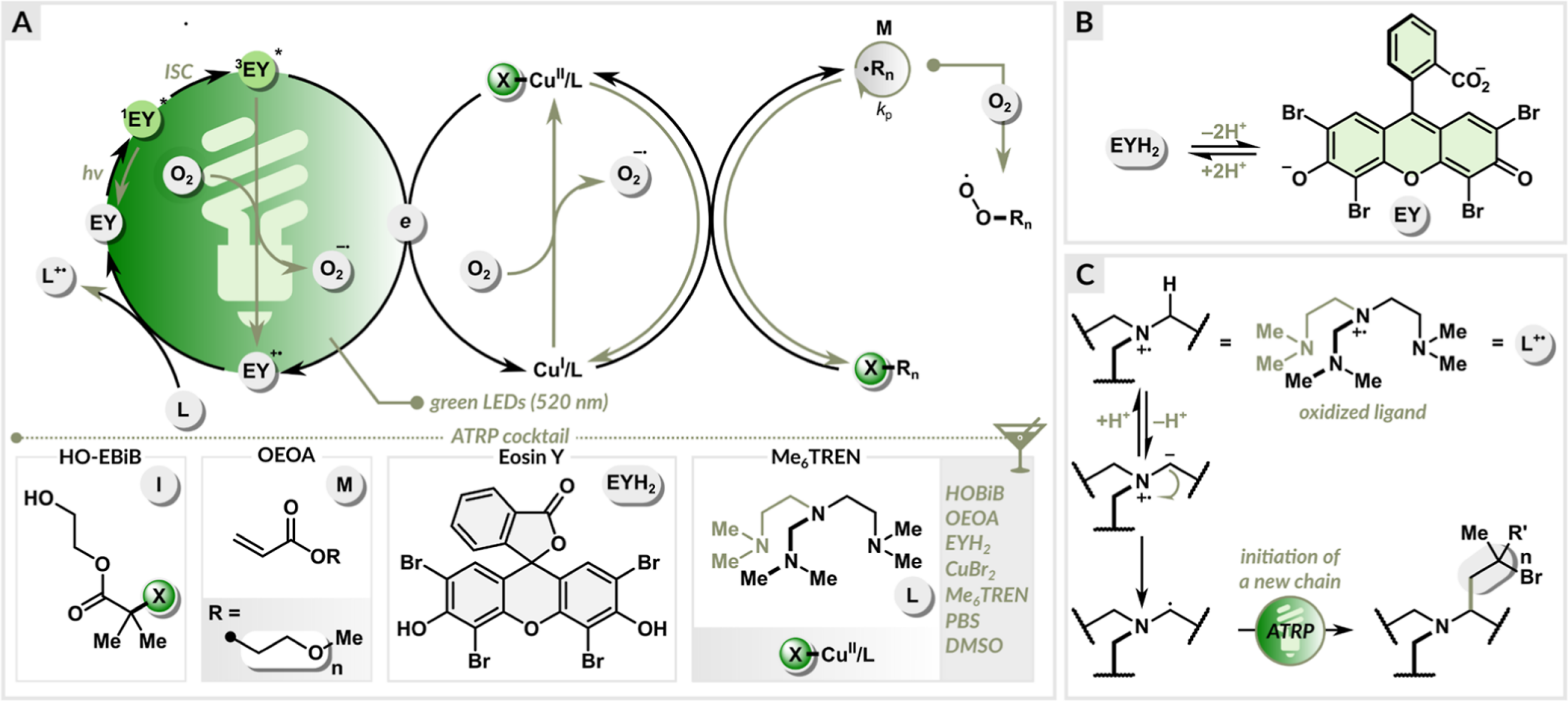
(a) Proposed mechanism of EY/Cu-catalyzed ATRP. (b) Deprotonation of EYH_2_ in PBS solution. (c) Formation of new polymer chains.

Eosin Y is a xanthene dye commonly used as a photocatalyst in visible-light-mediated RDRP techniques,^47,80^ particularly in photoinduced electron transfer RAFT (PET-RAFT) polymerization.^94−98^ Recently, we developed green-light-induced ATRP with dual catalysis, where eosin Y was used in combination with a copper complex (Figure 2a).^24,99^ This method proved to be highly efficient, allowing rapid and well-controlled aqueous polymerization of oligo(ethylene oxide) methyl ether methacrylate without the need for deoxygenation. Herein, we expanded the application of this system by preparing various well-defined polyacrylates and acrylate-based protein-polymer hybrids in water under ambient conditions.

## Results and Discussion

### Initial Optimizations of Dual Catalytic ATRP of OEOA_480_ in Aqueous Media

Oligo(ethylene oxide) methyl ether acrylate (average *M*_n_ = 480, OEOA_480_) was used as the model monomer, 2-hydroxyethyl α-bromoisobutyrate (HO-EBiB) as the initiator, eosin Y (EYH_2_) as the organic photoredox catalyst, and CuBr_2_/Me_6_TREN (Me_6_TREN = tris[2-(dimethylamino)ethyl]amine) as the deactivator (Figure 2a). Polymerizations were performed in open vials using an EvoluChem photoreactor (520 nm, 25.0 mW/cm^2^). Phosphate-buffered saline (PBS) with DMSO (10% *v*/*v*) was used as the reaction medium to ensure benign conditions and suppress the dissociation of the [X–Cu^II^/L]^+^ deactivator.^85,100,101^ Furthermore, the neutral form of eosin Y adopts a spirocyclic structure (EYH_2_), which impedes the absorption of green light.^102^ Using PBS results in the deprotonation of EYH_2_ and the formation of the anionic eosin Y (EY) with an opened ring (Figure 2b), exhibiting high photocatalytic activity. Table 1 shows the results of the polymerization of OEOA_480_ and the effect of different components of dual catalysis on the ATRP performance.

**Table 1:** Optimization of EY/Cu-Catalyzed ATRP of OEOA_480_.^a^

Entry	[HO-EBiB]	[CuBr_2_]	[Ligand]	[EYH_2_]	bConv. (%)	*M*_n,th_	c*M*_n,abs_	d*M*_n,app_	d*D̵*
1	1.0	0.2	0.6	0	3	2 900	n.d.	n.d.	n.d.
2	1.0	0	0.6	0.01	78	74 900	312 000	185 200	1.38
3	1.0	0.2	0.6	0.01	94	90 400	48 100	34 300	1.13
4	0	0.2	0.6	0.01	25	24 300	299 100	178 300	1.86
5	1.0	0.2	0.2	0.01	0	0	n.d.	n.d.	n.d.
6	1.0	0.2	0.3	0.01	76	72 900	75 400	51 400	1.14
7^e^	1.0	0.2	0.2	0.01	66	63 500	54 100	38 100	1.27
8^f^	1.0	0.2	0.6	0.01	5	4 900	10 900	9 000	1.22
9	1.0	0.4	0.6	0.01	45	43 200	73 000	50 000	1.17
10	1.0	0.2	0.3	0.005	75	72 200	73 200	50 100	1.18
11	1.0	0.1	0.3	0.005	82	78 700	76 700	52 200	1.25

a Reactions conditions: [OEOA_480_]/[HO-EBiB]/[EYH_2_]/[CuBr_2_]/[Me_6_TREN] = 200/1/x/x/x, [OEOA_480_] = 300 mM, in PBS with DMSO (10% *v*/*v*), irradiated for 30 min under green LEDs (520 nm, 25.0 mW/cm^2^) in an open HPLC vial.

b Monomer conversion was determined by using ^1^H NMR spectroscopy.

c Molecular weight (*M*_n,abs_) was determined by Mark–Houwink calibration (see the Supporting Information).

d Molecular weight (*M*_n,app_) and dispersity (*D̵*) were determined by SEC analysis (DMF as eluent) calibrated with poly(methyl methacrylate) standards.

e With an additional 0.3 equiv of TEAO.

f With TPMA ligand.

In the control experiment without EYH_2_, ^1^H NMR measurement showed that no polymer was formed after 30 min of green light irradiation (Table 1, entry 1), while in EY-catalyzed O-ATRP, 78% monomer conversion was achieved, but size exclusion chromatography (SEC) analysis revealed a moderate dispersity (*D̵*) of 1.38 and a significant deviation from the theoretical molecular weight value (*M*_n,th_ = 74 900, *M*_n,abs_ = 312 000, Table 1, entry 2). In contrast, the use of EYH_2_ and CuBr_2_ in the presence of excess Me_6_TREN ligand enabled rapid and well-controlled polymerization (*D̵* = 1.13), resulting in 94% monomer conversion within 30 min, despite the reaction vessel being open to the air (Table 1, entry 3). However, the molecular weight (*M*_n,abs_ = 48 100) of the polymer was much lower than the theoretical value (*M*_n,th_ = 90 400). In addition, under the same conditions, polymerization without the HO-EBiB initiator led to a 25% monomer conversion with high *M*_n,abs_ of 299 100 (Table 1, entry 4), indicating the photogeneration of new polymer chains. The Me_6_TREN ligand can bind to copper cations and, when used in excess, acts as a sacrificial electron donor, reducing EY^•+^ (Figure 1a). Electron transfer from the nitrogen atom of the Me_6_TREN ligand to EY^•+^ results in the formation of EY and the amine radical cation (R_3_N^•+^). The oxidized Me_6_TREN ligand, upon deprotonation,^103^ forms a radical that can reduce the Cu^II^/L or initiate a polymer chain (Figure 2c).

Further experiments were carried out to diminish the formation of new polymer chains and thus improve the agreement between the theoretical and apparent molecular weights. First, the amount of Me_6_TREN was decreased using a molar ratio of [CuBr_2_]/[Me_6_TREN] = 0.2/0.2. As expected, no polymerization occurred within 30 min (Table 1, entry 5), as there was no free ligand remaining that could reduce the oxidized EY (Figure 2a). Therefore, the amount of Me_6_TREN was slightly increased ([CuBr_2_]/[Me_6_TREN] = 0.2/0.3). This greatly improved the control over molecular weight and its distribution (conv. = 76%, *M*_n,th_ = 72 900, *M*_n,abs_ = 75 400, *D̵* = 1.14) (Table 1, entry 6).

Since Me_6_TREN is a relatively expensive compound, triethanolamine (TEOA) was used as a sacrificial electron donor at a molar ratio of [CuBr_2_]/[Me_6_TREN]/[TEOA] = 0.2/0.2/0.3, but a lower monomer conversion (66%) and no improvement in the agreement of the theoretical and experimental molecular weight were observed (Table 1, entry 7). Also, the use of less active tris(2-pyridylmethyl) amine (TPMA) ligand ([CuBr_2_]/[TPMA] = 0.2/0.6) resulted in a significant decrease in the rate of the polymerization (Table 1, entry 8).

Finally, the effect of CuBr_2_ and EYH_2_ concentrations on the polymerization performance of OEOA_480_ was investigated (Table 1, entries 9–11). Increasing the amount of CuBr_2_ twofold resulted in lower monomer conversion (45%) with no significant improvement over polymerization control (Table 1, entry 9). In contrast, decreasing the amount of EYH_2_ to 25 ppm (relative to the monomer) while maintaining the optimal Cu concentration (0.3 mM) yielded faster kinetics with no significant impact on the dispersity (Table 1, entry 10), whereas decreasing the concentration of Cu to 0.15 mM resulted in higher dispersity (Table 1, entry 11).

### Kinetics of EY/Cu-Catalyzed ATRP

**Figure 3:**
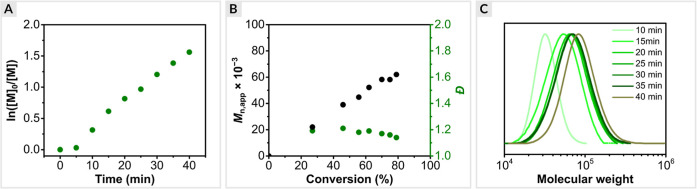
Kinetics of the optimized EY/Cu-catalyzed ATRP of OEOA_480_. (a) First-order kinetic plot. (b) Evolution of molecular weight and molecular weight distribution with conversion, (c) and molecular weight distribution evolution with time. Reaction conditions: [OEOA_480_]/[HO-EBiB]/[EYH_2_]/[CuBr_2_]/[Me_6_TREN] = 200/1/0.01/0.2/0.3 in PBS with DMSO (10% *v*/*v*), irradiated under green LEDs (520 nm, 25.0 mW/cm^2^) in an open vial with stirring at 500 rpm.

Photoinduced ATRP was performed under the optimized conditions ([OEOA_480_]/[HO-EBiB]/[EYH_2_]/[CuBr_2_]/[Me_6_TREN] = 200/1/0.01/0.2/0.3). The samples were taken at regular intervals, quenched with 1,4-bis(3-isocyanopropyl)piperazine,^104^ and then analyzed by ^1^H NMR and SEC. EY/Cu-catalyzed ATRP of OEOA_480_ exhibited first-order kinetics with a short induction period of 5 min, followed by a rapid polymerization, reaching 78% monomer conversion within 40 min (Figures 3a and S4). The short induction period was ascribed to the time required to remove oxygen from the polymerization solution. A good agreement between theoretical and experimental molecular weights was observed. In addition, SEC traces revealed the molecular weights increased as a function of monomer conversion and the dispersity remained low (*D̵* < 1.2) during the polymerization (Figure 3b).

### Temporal Control

**Figure 4:**
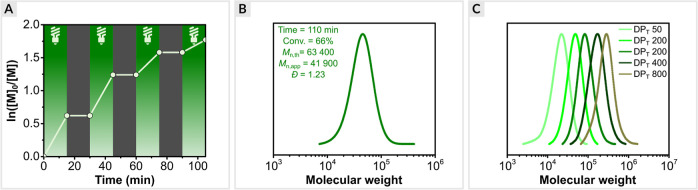
(a) Temporal control in EY/Cu-catalyzed ATRP of OEOA_480_. (b) SEC analysis of the resulting polymer after 110 min (four cycles of light on/off periods). (c) SEC analysis of poly(OEOA_480_) with varying targeted DP.

Next, the optimized polymerization of OEOA_480_ was carried out in an open vial with intermittent light exposure (Figure 4a). The dual catalytic system exhibited high temporal control. Irradiation with green light-triggered and sustained polymerization while turning off the light almost completely stopped it. During the dark period, the rapid oxidation of the Cu^I^/L activator to its inactive form halted polymerization. Temporal control in ATRP is typically achieved with low Cu concentrations, but this leads to poor control over polymerization.^105,106^ The final polymer showed monomodal SEC trace and low dispersity (*D̵* = 1.23, Figure 4b), indicating that intermittent light irradiation does not impair the control over polymerization.

### Varying Targeted Degrees of Polymerization of OEOA_480_

In order to change the target degree of polymerization (DP_T_), the concentration of HO-EBiB was varied (6.0–0.375 mM), keeping the concentrations of OEOA_480_ (300 mM), EYH_2_ (15 μM), CuBr_2_ (0.3 mM), and Me_6_TREN (0.45 mM) constant (Table 2). In all cases, the monomer conversions reached ≥70% within 40 min. The synthesized polymers showed a narrow molecular weight distribution (1.17 ≤ *D̵* ≤ 1.23) for a wide targeted DP range (50–800). Symmetrical SEC traces with no detectable tailing or high molecular weight shoulders demonstrated the robustness of the dual EY/Cu catalysis in the synthesis of poly(OEOA_480_) over a wide range of molecular weights (Figure 4c).

**Table 2:** EY/Cu-Catalyzed ATRP of OEOA_480_ With Varying Degrees of Polymerization^a^

entry	[M]/[I]/[EYH_2_]/[CuBr_2_]/[Me_6_TREN]	[*I*] (mM)	bconv. (%)	*M*_n,th_	c*M*_n,abs_	d*M*_n,app_	d*D̵*
1	50/1/0.0025/0.05/0.075	6.0	89	21 400	19 200	19 200	1.22
2	100/1/0.005/0.1/0.15	3.0	81	38 900	25 300	29 200	1.17
3	200/1/0.01/0.2/0.3	1.5	76	73 200	73 900	50 500	1.17
4	400/1/0.02/0.4/0.6	0.75	73	140 200	141 600	90 800	1.23
5	800/1/0.04/0.8/1.2	0.375	70	268 800	240 600	146 500	1.20

a Reactions conditions [M] = [OEOA_480_] = 300 mM, [*I*] = [HO-EBiB] = 6.0–0.375 mM, [EYH_2_] = 15 μM, [CuBr_2_] = 0.3 mM, [Me_6_TREN] = 0.45 mM in PBS with DMSO (10% *v*/*v*), irradiated for 40 min under green LEDs (520 nm, 25.0 mW/cm^2^) in an open vial with stirring at 500 rpm.

b Monomer conversion was determined by using ^1^H NMR spectroscopy.

c Molecular weight (*M*_n,abs_) was determined by Mark–Houwink calibration (see the Supporting Information).

d Molecular weight (*M*_n,app_) and dispersity (*D̵*) were determined by SEC analysis (DMF as eluent) calibrated by poly(methyl methacrylate) standards.

### Monomer Scope of Photoinduced EY-Cu-Catalyzed ATRP

The monomer scope was then further expanded to 2-hydroxyethyl acrylate (HEA), 2-(methylsulfinyl)ethyl acrylate (MSEA), and zwitterionic carboxy betaine acrylate (CBA) (Table 3). Under the optimized conditions ([M]/[HO-EBiB]/[EYH_2_]/[CuBr_2_]/[Me_6_TREN] = 200/1/0.01/0.2/0.3), all monomers reached >50% conversion within 40 min. Moreover, the SEC traces remained monomodal and showed a narrow molecular weight distribution (Figures S5 and S6).

**Table 3:** EY-Cu-Catalyzed ATRP of Various Hydrophilic Acrylate Monomers^a^

entry	monomer	time (min)	bconv. (%)	c*M*_n,th_	c*M*_n,app_	c*D̵*
1	HEA	30	74	17 400	24 600	1.16
2	MSEA	30	54	17 700	32 200	1.19
3^d^	CBA	40	78	34 490	36 800	1.22

a Reactions conditions: [M]/[HO-EBiB]/[EYH_2_]/[CuBr_2_]/[Me_6_TREN]: 200/1/0.01/0.2/0.3. [M] = 300 mM, [HO-EBiB] = 1.5 mM, [EYH_2_] = 15 μM, [CuBr_2_] = 0.3 mM, [Me_6_TREN] = 0.45 mM in PBS with DMSO (10% *v*/*v*), irradiated under green LEDs (520 nm, 25.0 mW/cm^2^) in an open vial with stirring at 500 rpm.

b Monomer conversion was determined by using ^1^H NMR spectroscopy.

c Molecular weight and dispersity (*D̵*) were determined by SEC analysis (DMF as eluent) calibrated by poly(methyl methacrylate) standards.

d Absolute molecular weight and dispersity (*D̵*) were determined by SEC-MALS analysis (DPBS as eluent).

### In Situ Chain Extension and Block Copolymerization

**Figure 5:**
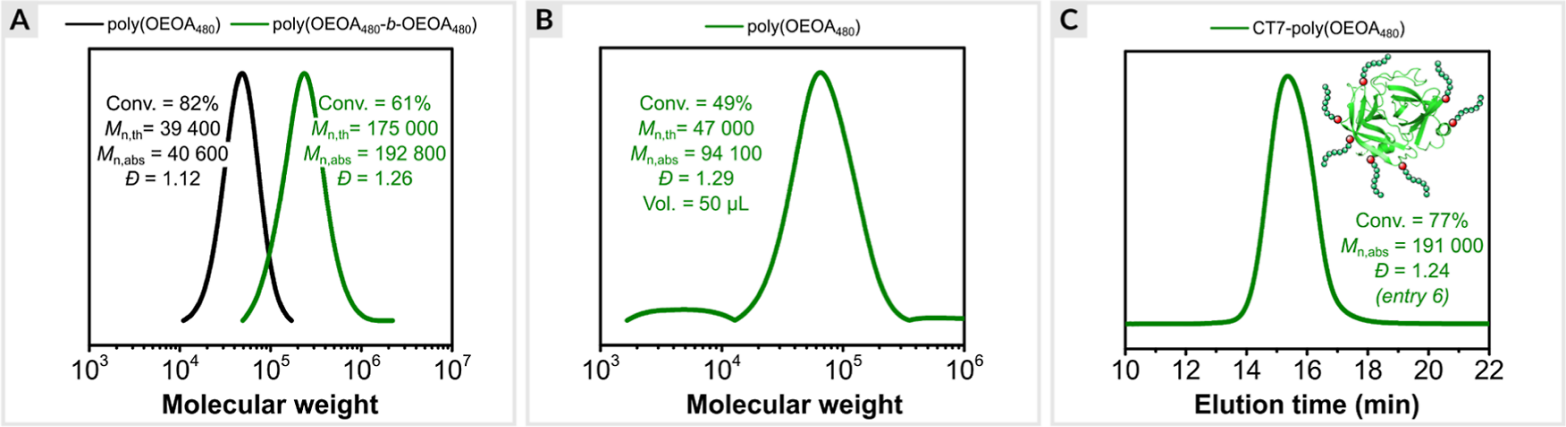
(a) In situ chain extension of poly(OEOA_480_). (b) Small-volume ATRP. (c) Synthesis of an acrylate-based protein-polymer hybrid.

The end-group fidelity was evaluated by in situ chain extension of poly(OEOA_480_) with different acrylate monomers. First, poly(OEOA_480_) was synthesized with [OEOA_480_]/[HO-EBiB]/[EYH_2_]/[CuBr_2_]/[Me_6_TREN] molar ratios of 100/1/0.005/0.1/0.15 (conv. = 82%, *M*_n,abs_ = 40 600, *D̵* = 1.12). The aliquot of the post-polymerization mixture was then used without further purification to prepare the second polymer block with OEOA_480_ monomer (DP_T_ = 500). The reaction was irradiated with a green light for 40 min, yielding a chain-extended polymer (conv. = 61%, *M*_n,app_ = 192 800, *D̵* = 1.26). The SEC analysis showed a clear shift of the polymer trace toward higher molecular weights without any shoulder or tailing at lower molecular weights, indicating high end-group fidelity (Figure 5a). In addition, well-controlled poly(OEOA_480_-*b*-HEA) and poly(OEOA_480_-*b*-MSEA) were obtained in a similar manner (Figure S7).

### Small-Volume ATRP

ATRP at μL-scale is an attractive method for grafting from expensive initiators such as therapeutic proteins and DNA.^107−109^ However, conventional ATRP at small volumes is challenging because rigorous deoxygenation can lead to solvent loss, affecting polymerization kinetics. In addition, inert gas sparging or freeze–pump–thaw degassing can cause a loss of enzymatic activity of protein-polymer hybrids.^20^

The polymerization of OEOA_480_ ([OEOA_480_]/[HO-EBiB]/[EYH_2_]/[CuBr_2_]/[Me_6_TREN] = 200/1/0.01/0.2/0.3) was performed in glass insert at a volume of 50 μL (conv. = 49%, *M*_n,abs_ = 94 100, *D̵* = 1.29, Figure 5b). The increased diffusion of oxygen into the system may explain the lower monomer conversion and slightly higher dispersity compared to polymerization at a larger volume. Nevertheless, this result indicates that this technique can be used in high-throughput, combinatorial polymer synthesis.^110,111^

### Synthesis of Protein-Polymer Hybrids

Proteins are commonly used as catalysts and therapeutics.^112,113^ Unfortunately, most proteins have limited stability and are easily denatured by heat, solvents, and inadequate salt concentrations.^114^ In addition, some proteins are inherently immunogenic.^115^ These problems can be overcome by attaching polymers to the surface of proteins using the grafting-from or grafting-to approach.^116−119^ Protein-polymer hybrids (PPHs) can exhibit greater stability and reduced immunogenicity.^120^ Examples of polyacrylamide- and polymethacrylate-based PPHs are abundant in the literature.^19,121^ In contrast, the synthesis of acrylate-based PPHs remains relatively underexplored, and the few available examples in the literature are limited to the use of RAFT polymerization or ATRP methods.^58,122−125^

The EY/Cu-catalyzed ATRP was extended to synthesize acrylate-based PPHs by grafting from approach. First, α-chymotrypsin macroinitiators with 7 and 12 ATRP initiator sites (CT-7 and CT-12, respectively) were synthesized according to a previously reported method.^126^ The protein–polymer bioconjugates were then prepared by grafting poly(OEOA_480_) chains from the surface of CT-7 or CT-12 (Table 4). All polymerizations were carried out at 15–18 °C in a Lumidox photoreactor (527 nm, 125 mW/cm^2^) to preserve the activity of chymotrypsin. Initial studies began by polymerizing OEOA_480_ at 300 mM concentration (DP_T_ = 200), using CT-12 as the macroinitiator and dual catalysis with [EYH_2_]/[CuBr_2_]/[Me_6_TREN] molar ratios of 0.01/0.2/0.3. After 3 h of green light irradiation, the conversion of OEOA_480_ was 38% (Table 4, entry 1). The slower reaction rate compared to the homopolymerization of OEOA_480_ was attributed to the lower temperature. The successful synthesis of acrylate-based PPH was demonstrated with SEC equipped with a multi-angle light scattering (MALS) detector, where a monomodal trace was observed with a higher molecular weight compared to native chymotrypsin. However, the dispersity value for the obtained PPH was high (*D̵* = 1.72). Attempts to further improve the control over the polymerization by decreasing monomer concentration, targeting lower DP or preventing oxygen diffusion by closing the reaction vessel only led to higher monomer conversion, while dispersity was not significantly improved (Table 4, entries 2–4). Broad molecular weight distribution could be explained by the enhanced intra- and inter-radical termination reactions due to increased viscosity. Therefore, we used a protein macroinitiator with a lower number of initiating sites (CT-7), decreased the OEOA_480_ concentration to 100 mM, and lowered the DP_T_ to 50 (Table 4, entries 5–7). This resulted in significantly better control of molecular weight distribution (*D̵* = 1.15) (Table 4, entry 5). Importantly, decreasing Cu^II^/L concentration did not lead to a loss of ATRP control. The optimized reaction conditions were also used to synthesize PPHs with higher DPs (100 and 200), giving better control compared to grafting from CT-12 (Table 4, entries 6 and 7, Figure 5c). These results demonstrate that various acrylate-based PPHs can be synthesized by EY/Cu-catalyzed ATRP using low-energy green light and a straightforward reaction setup without any rigorous deoxygenation.

**Table 4:** Synthesis of PPHs by EY/Cu-Catalyzed ATRP^a^

entry	protein	[M]/[EYH_2_]/[CuBr_2_]/[L]	[*M*] (mM)	vial	time (h)	bconv. (%)	c*M*_n,abs_ × 10^3^	c*D̵*
1	CT-12	200/0.01/0.2/0.3	300	open	3	38	280	1.72
2	CT-12	200/0.01/0.2/0.3	300	closed	3.5	38	201	1.78
3	CT-12	100/0.01/0.2/0.3	300	open	3.5	72	231	1.78
4	CT-12	100/0.01/0.2/0.2	100	closed	2	55	121	1.61
5	CT-7	50/0.005/0.1/0.1	100	closed	1.3	34	72	1.15
6	CT-7	100/0.01/0.2/0.2	100	closed	3	77	191	1.24
7	CT-7	200/0.02/0.4/0.4	100	closed	3	71	224	1.56

a Reactions conditions: [OEOA_480_]/[CT-12 or CT-7]/[EYH_2_]/[CuBr_2_]/[Me_6_TREN] = 50–200/x/x/x/x, [OEOA_480_] = 300 or 100 mM, in PBS with DMSO (10% *v*/*v*), irradiated under green LEDs (527 nm, 125 mW/cm^2^) in a HPLC vial.

b Monomer conversion was determined by using ^1^H NMR spectroscopy.

c Molecular weight (*M*_n,abs_) and dispersity (*D̵*) were determined by SEC analysis (DPBS as eluent) with MALS detectors.

Finally, the enzymatic activity of PPH (Table 4, entry 6) was analyzed by measuring Michaelis–Menten parameters using *N*-Succinyl-Ala-Ala-Pro-Phe-*p*-nitroanilide (Suc-AAPF-pNA) as a substrate for chymotrypsin (CT).^127^ The activity of the PPH was ca. 4-fold lower than native CT (Table S3). Such loss in the activity of CT was attributed to the shielding of enzyme active sites by poly(OEOA_480_) rather than polymerization conditions, as previously reported.^128^

## Conclusions

EY/Cu-catalyzed ATRP was used to prepare a variety of well-defined water-soluble acrylates under bio-relevant conditions using low-energy green light. The dual catalysis proved to be highly efficient, allowing the synthesis of poly(OEOA_480_) in the open air within 40 min. The synthesized polymers showed narrow molecular weight distribution (1.17 ≤ *D̵* ≤ 1.23) for a wide targeted DP range (50–800) despite the use of Cu^II^/Me_6_TREN and eosin Y at ppm levels. The preserved chain end functionality was confirmed by in situ chain extensions. In addition, the optimized conditions also enabled controlled polymerization of 2-hydroxyethyl acrylate, 2-(methylsulfinyl)ethyl acrylate), and zwitterionic carboxy betaine acrylate. Importantly, the method allowed the synthesis of acrylate-based protein-polymer hybrids from chymotrypsin with 7 and 12 initiator sites using a straightforward reaction setup without any rigorous deoxygenation. This work greatly expands the family of monomers that can be polymerized or grafted from proteins using photo-ATRP under ambient conditions.

## Supporting Information Available

Details of experimental procedures and additional control experiments and polymerization results (PDF)
