# Supplementary material for: Fully Oxygen-Tolerant Visible-Light-Induced ATRP of Acrylates in Water: Toward Synthesis of Protein-Polymer Hybrids
Source: Macromolecules. 2023 Feb 20;56(5):2017–26. doi: 10.1021/acs.macromol.2c02537 (PMC10019465; doi:10.1021/acs.macromol.2c02537)
Supplement: Supplementary file 1 — ma2c02537_si_001.pdf [file ma2c02537_si_001.pdf]

Fully Oxygen-Tolerant Visible-Light-Induced ATRP of Acrylates in Water: Toward Synthesis of Protein-Polymer Hybrids

Kriti Kapil,† Arman Moini Jazani,† Grzegorz Szczepaniak, Hironobu Murata, Mateusz Olszewski, and Krzysztof Matyjaszewski*

Department of Chemistry, Carnegie Mellon University, 4400 Fifth Avenue, Pittsburgh, PA 15213, United States.

† These authors contribute equally.

Table of Contents

Experimental Details 3
Materials 3
Instrumentation 3
Nuclear Magnetic Resonance (NMR) 3
Size Exclusion Chromatography 4
Size Exclusion Chromatography with Multi-Angle Light Scattering (SEC-MALS) 4
dn/dc value of poly($OEOA_{480}$) 4
Determination of Mark-Houwink Parameters of $OEOA_{480}$ in DMF 5
Monomer synthesis 6
Synthesis of 3-[[2-(acryloyloxy) ethyl] dimethylammonio] propionate (CBA) 6
Polymerizations 7
EY/Cu-catalyzed ATRP of $OEOA_{480}$ (Table 1) 7
Kinetics of EY/Cu-catalyzed ATRP (Figure 3) 9
Temporal control (Figure 4a) 10
EY/Cu-catalyzed ATRP of $OEOA_{480}$ with varying degrees of polymerization (Table 2) 10
Expanding the scope to other hydrophilic acrylates (Table 3) 11
Synthesis of block copolymers by *in-situ* chain extension (Figure 5a) 12
Synthesis of linear poly($OEOA_{480}$) (DP_T = 100) 12
In-situ chain extension of linear poly($OEOA_{480}$) (DP_T = 100) 12
Synthesis of protein-polymer hybrids 13
Synthesis and characterization of CT macroinitiators with 7 and 12 ATRP initiators 13
General procedure for the synthesis of CT-poly($OEOA_{480}$) biohybrids (Table 4) 14
CT-7-poly($OEOA_{480}$) biohybrid enzymatic activity 14

Experimental Details

Materials

All chemicals were purchased from commercial sources and used as received unless otherwise noted. Tris(2-pyridylmethyl) amine (TPMA, 99%), tris[2-(dimethylamino) ethyl] amine (Me_6TREN, 99%) were purchased from *AmBeed*. Eosin Y (EYH_2, 99%), copper (II) bromide ($CuBr_2$, 99.99%), 2-hydroxyethyl *α*-bromoisobutyrate (HO-EBiB, 95%), 1,4-bis(3-isocyanopropyl) piperazine (QA), triethanolamine (TEOA, ≥99.0%), α-chymotrypsin (CT) from bovine pancreas were purchased from *Sigma-Aldrich*. 10X PBS ware purchased from *Thermo Fisher Scientific*. Oligo(ethylene oxide) methyl ether acrylate (average M_n = 480, $OEOA_{480}$), 2-hydroxyethyl acrylate (HEA) were purchased from *Sigma-Aldrich* and passed through a column of basic alumina to remove inhibitor prior to use. Water (HPLC grade) and dimethyl sulfoxide (DMSO, ≥99.7%) were purchased from *Fisher*. *N*-succinyl-L-Ala-L-Ala-L-Pro-L-Phe-p-nitroanilide (sucAAPFpNA) was purchased from *Bachem*. 2-(methylsulfinyl) ethyl acrylate (MSEA) and *N*-2-bromo-2-methylpropionyl-*β*-alanine *N*'-oxysuccinimide ester (NHS-Br) were synthesized according to the previous publications.[1]

Instrumentation

Nuclear Magnetic Resonance (NMR)

^{1}H NMR spectra were recorded on *Bruker* Avance III 500 MHz spectrometers with D_2O or DMSO-d_6 as the solvent.

[1] S. Li, H. S. Chung, A. Simakova, Z. Wang, S. Park, L. Fu, D. Cohen-Karni, S. Averick and K. Matyjaszewski, *Biomacromolecules*, **2017**, 18, 475–482.

Size Exclusion Chromatography

SEC measurements of polymers were performed using an Agilent GPC equipped with a RI detector and PSS columns (Styrogel 10^5, 10^3, 10^2 Å) with DMF as an eluent at 50 °C and the flow rate of 1 mL/min. Linear poly(methyl methacrylate) standards were used for calibration.

Size Exclusion Chromatography with Multi-Angle Light Scattering (SEC-MALS)

SEC-MALS measurements of zwitterionic polyacrylate and bioconjugates were performed using Agilent SEC system (*Agilent*, 1260 Infinity II) coupled with MALS, DLS, UV, Viscometer and RI detectors (*Wyatt Technology*, USA). Measurements were performed using *Waters* Ultra hydrogel Linear column with 1X DPBS as an eluent at room temperature and the flow rate of 0.5 mL/min.

dn/dc value of poly($OEOA_{480}$)

The dn/dc value of poly($OEOA_{480}$) in DMF with 0.05 M LiBr at 50 °C was determined using the refractive index (RI) detector Waters 2414 (Milford, MA). Five samples of poly($OEOA_{480}$) ($M_{n,app}$ = 51 500, $Đ$ = 1.16) purified by dialysis were prepared using the standard dilution method (Table S1). Using syringe pump Chemyx, Fusion 4000 (Stafford, TX), the DMF was injected into the RI detector at a flow rate of 0.1 mL/min until it stabilized. Then 1 mL of polymer sample was injected at a constant flow rate, and RI values was measured (Table S1). Each sample was repeated three times, and the averaged RI values were used to obtain a calibration plot, the slope of which gives the dn/dc value of poly($OEOA_{480}$) as 0.047 mL/g.

Table S1. Measured RI values for different concentrations of poly($OEOA_{480}$).

Entry	Conc. (mg/mL)	Measured RI
1	0.625	8.91
2	1.25	45.3
3	2.5	113.6
4	5	224.5
5	10	448.1

Determination of Mark-Houwink Parameters of $OEOA_{480}$ in DMF

Mark-Houwink parameters (K = 76.9, a = 0.482) for poly($OEOA_{480}$) in DMF with 0.05 M LiBr at 50 °C were determined according to the previously reported method.[2]

Table S2. $M_{n,app}$ vs. $M_{n,MALS}$ of poly($OEOA_{480}$) samples.

Entry	$^{a}M_{n,app}$	$Log(M_1)$	$^{b}M_{n,MALS}$	$Log(M_2)$
1	14 300	4.15	14 500	4.16
2	21 700	4.34	24 100	4.38
3	31 200	4.49	38 400	4.58
4	32 000	4.51	48 000	4.68
5	37 400	4.57	49 800	4.70
6	48 100	4.68	67 400	4.83
7	54 400	4.74	84 600	4.93
8	82 100	4.91	106 000	5.03
9	84 300	4.93	124 000	5.09
10	89 700	4.95	143 400	5.16
11	105 000	5.02	153 300	5.19
12	137 100	5.14	221 700	5.35
13	148 400	5.17	222 500	5.35
14	160 300	5.20	237 100	5.37
15	218 00	5.34	344 200	5.54
16	225 700	5.35	297 300	5.47
17	240 600	5.38	377 700	5.58
18	287 200	5.46	465 700	5.67

[a)]Molecular weight ($M_{n,app}$) was determined by SEC analysis (DMF as eluent) calibrated with poly(methyl methacrylate) standards. [b)]Molecular weight ($M_{n,MALS}$) was determined by SEC-MALS analysis (DMF as eluent, dn/dc = 0.047 mL/g).

[2] G. Szczepaniak, J. Jeong, K. Kapil, S. Dadashi-Silab, S. S. Yerneni, P. Ratajczyk, S. Lathwal, D. J. Schild, S. R. Das and K. Matyjaszewski, *Chem. Sci.*, **2022**, 13, 11540−11550.

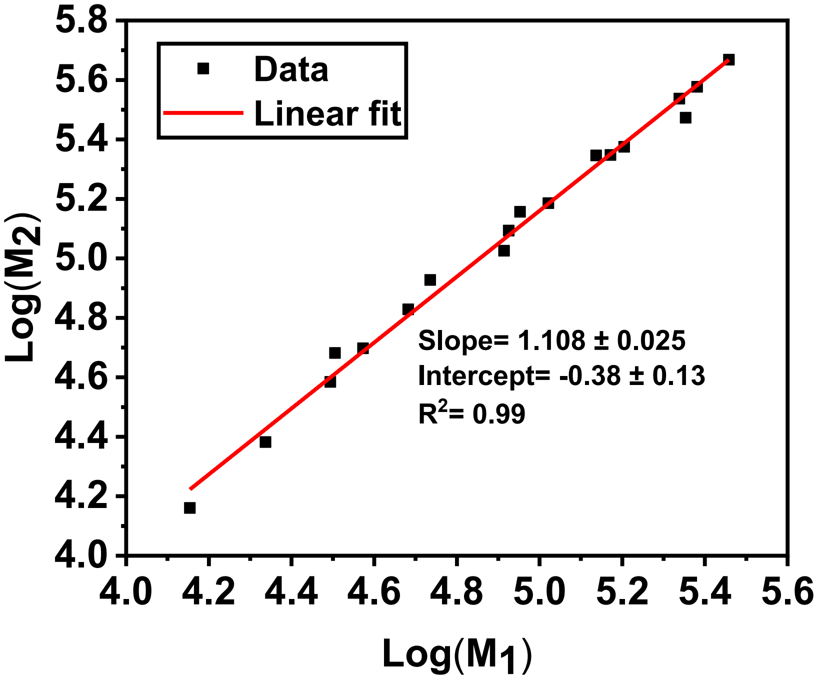

Figure S1. Mark-Houwink fits to log(M_2) vs. log(M_1).

Monomer synthesis

Synthesis of 3-[[2-(acryloyloxy) ethyl] dimethylammonio] propionate (CBA)

To a solution of 2-(dimethylamino) ethyl acrylate (2.9 g, 20 mmol) in dry THF (100 mL), *β*-propiolactone (1.0 mL, 16 mmol) was slowly added and the mixture was stirred at 4 °C for 20 h. The precipitate was filtered, washed with dry THF and ether, and dried in vacuo. 3-[[2-(acryloyloxy) ethyl] dimethylammonio] propionate was obtained as white powder (2.2 g, 52%). ^{1}H NMR (500 MHz, D_2O) δ 2.67 (t, *J* = 8.0 Hz, 2H, $N^+CH_2CH_2COO^-$), 3.12 (s, 6H, $N^+(CH_3)_2$), 3.61 (t, *J* = 8.0 Hz, 2H, $N^+CH_2CH_2COO^-$), 3.71 – 3.73 (m, 2H, $C{=}OCH_2CH_2N^+$), 4.58 – 4.60 (m, 2H, $C{=}OCH_2CH_2N^+$), 5.98 (d, *J* = 10.5 Hz, 1H, vinyl), 6.16 (dd, J = 10.5 and 17.5 Hz, 1H, vinyl) and 6.40 (d, *J* = 17.5 Hz, 1H, vinyl) ppm.

Polymerizations

Polymerizations were carried out in the EvoluChem PhotoRedOx Box™ device purchased from *HepatoChem* equipped with green LEDs (λ = 525 nm, *Kessil*) (Figure S2). The polymerizations were carried out in 2 mL clear HPLC autosampler vials (optimization of reaction conditions) or in clear 1-dram vials (12/96 mm) with a stirring rate of 500 rpm, open to the air. Synthesis of biohybrids was carried out using the Lumidox® photoreactor equipped with green LEDs (525 nm, 125 mW/cm^2) and a cooling system.

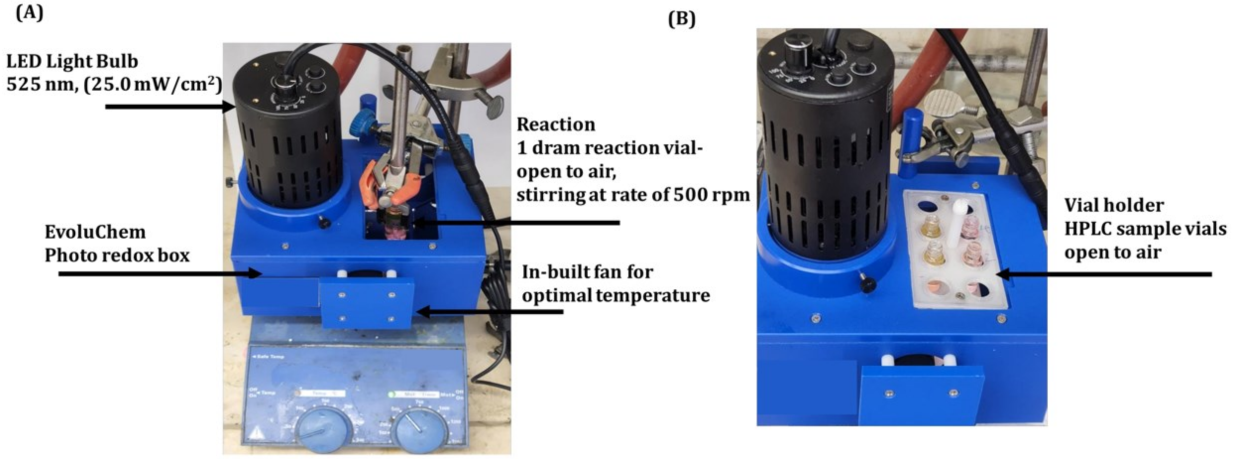

Figure S2. Polymerization set-up in the Evoluchem Photoredox box (A) General set-up for the polymerizations (B) High-throughput polymerizations (Table 1).

EY/Cu-catalyzed ATRP of OEOA$_{480}$ (Table 1)

Prior to polymerizations, stock solutions of alkyl halide initiator HO-EBiB (15.8 mg in 1.0 mL DMSO), $CuBr_2$ (33.5 mg in 20.0 mL DMSO), Me_6TREN (5.18 mg in 1.0 mL DMSO), TPMA (13.1 mg in 1.0 mL DMSO), EYH_2 (0.97 mg in 1 mL DMSO), and TEOA (11.18 mg in 5.0 mL DMSO) were prepared.

In a 2 mL volumetric flask, 288 mg (0.6 mmol) OEOA$_{480}$ was weighed. $CuBr_2$ stock (80 µL), Me_6TREN stock (40 µL), HO-EBiB stock (40 µL), EYH_2 stock (20 µL), DMF (20 µL), and 10X PBS solution (200 µL) were then added. HPLC-grade water was used to make up the rest of the volume in the volumetric flask. The reaction mixture was transferred to a 2 mL autosampler GPC vial and placed on 8×2 ml vial holder (product no. HCK1006-01-018) inside the Evoluchem Photo redox

box (Figure S2B). The reaction vial was irradiated for 30 min under green LEDs (525 nm, 25.0 mW/cm^2). Samples were taken and analyzed by ^{1}H NMR and SEC techniques.

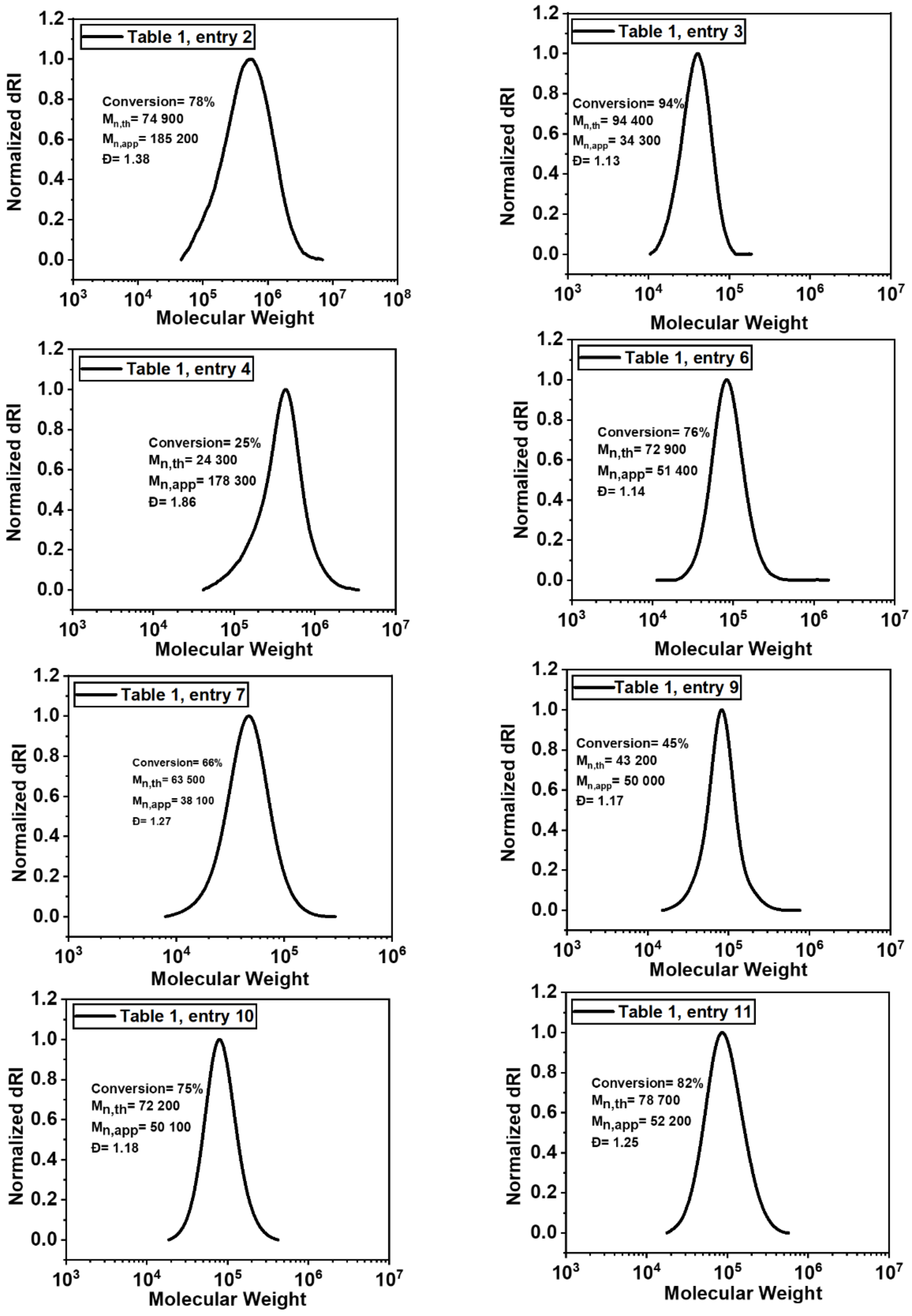

Figure S3. SEC traces for Table 1.

Kinetics of EY/Cu-catalyzed ATRP (Figure 3)

The ATRP cocktail (5 mL) was prepared according to the general procedure. The final concentrations were: $OEOA_{480}$ (300 mM), HO-EBiB (1.5 mM), EYH_2 (15 µM), $CuBr_2$ (0.3 mM), and Me_6TREN (0.45 mM). 50 µL of DMF was used as an internal standard. Then 4.4 mL of the ATRP cocktail ($[OEOA_{480}]/[HO\text{-}EBiB]/[EYH_2]/[CuBr_2]/[Me_6TREN]$ = 200/1/0.01/0.2/0.3) was added to a 1-dram (12/96 mm) vial equipped with a magnetic stir bar. The polymerization mixture in an uncapped vial was stirred at 500 rpm for 40 min under green LEDs (520 nm, 9.0 mW/cm^2). Samples were drawn at regular intervals and monitored by ^1H NMR in D_2O (Figure S4) and by SEC (Figure 2).

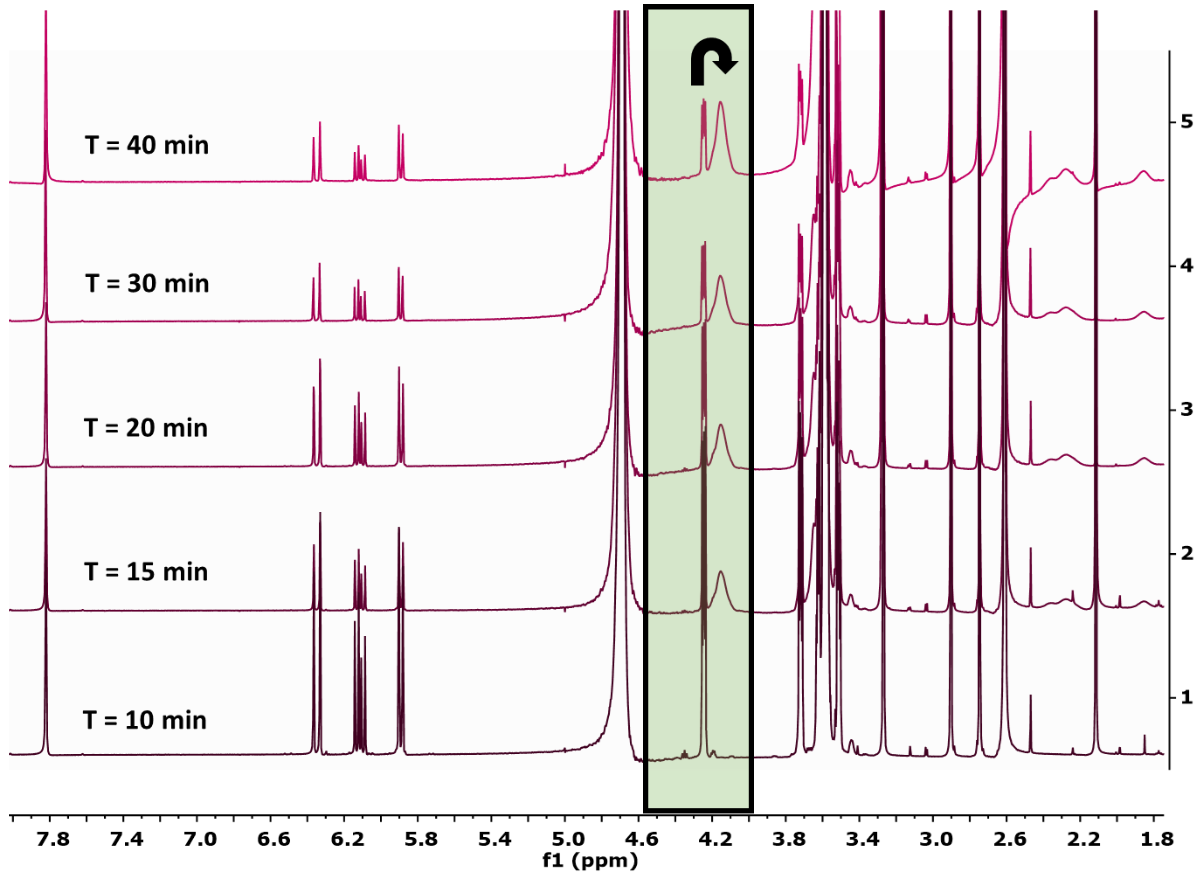

Figure S4. Overlapped ^1H NMR spectra showing kinetics of EY/Cu-catalyzed green-light induced ATRP of $OEOA_{480}$.

Temporal control (Figure 4a)

The ATRP cocktail (5 mL) was prepared according to the general procedure. The final concentrations were: $OEOA_{480}$ (300 mM), HO-EBiB (1.5 mM), EYH_2 (15 μM), $CuBr_2$ (0.3 mM), Me_6TREN (0.45 mM), DMSO (10% *v*/*v*). Then 4.4 mL of the ATRP cocktail ([$OEOA_{480}$]/[HO-EBiB]/[EYH_2]/[$CuBr_2$]/[Me_6TREN] = 200/1/0.01/0.2/0.3) was added to a 1-dram (12/96 mm) vial equipped with a magnetic stirrer. The polymerization mixture in an uncapped vial was stirred at 500 rpm under green LEDs (525 nm, 25.0 mW/cm^2), and the light was turned on/off at 15 min intervals. Samples (50 μL) were drawn out and quenched with 20 μL of 1,4-bis(3-isocyanopropyl)piperazine (10 mg/mL in D_2O) and then analyzed by ^1H NMR.[3]

EY/Cu-catalyzed ATRP of $OEOA_{480}$ with varying degrees of polymerization (Table 2)

The target degrees of polymerization (DP) were varied by adjusting the HO-EBiB concentration (6 mM–0.375 mM), while the concentrations of all the other components, such as $OEOA_{480}$ (300 mM), HO-EBiB (1.5 mM), EYH_2 (15 μM), $CuBr_2$ (0.3 mM), Me_6TREN (0.45 mM), DMSO (10% v/v) were kept constant. The HO-EBiB stock solution was prepared (63.3 mg in 1.0 mL DMSO, 300 mM), and using standard dilution procedure, other stock solutions of varying concentrations of HO-EBiB (150 mM, 75 mM, 37.5 mM, 18.75 mM) were prepared in DMSO. ATRP cocktails (5 mL) were prepared according to the general procedure by adding HO-EBiB stock solution (100 μL) at a different concentration to obtain the target DP (50, 100, 200, 400, and 800). Then 4.4 mL of the ATRP cocktail with targeted DP was added to a 1-dram (12/96 mm) vial equipped with a magnetic stir bar. The polymerization mixture in an uncapped vial was stirred at 500 rpm for 40 min under green LEDs (525 nm, 25.0 mW/cm^2). Samples were taken and analyzed by ^1H NMR and SEC techniques.

---

[3] G. Szczepaniak, J. Piątkowski, W. Nogaś, F. Lorandi, S. S. Yerneni, M. Fantin, A. Ruszczyńska, A. E. Enciso, E. Bulska, K. Grela and K. Matyjaszewski, *Chem. Sci.*, **2020**, 11, 4251–4262

Expanding the scope to other hydrophilic acrylates (Table 3)

The polymerization reaction mixtures were prepared according to the general procedure. The final concentrations were: monomer (300 mM), HO-EBiB (1.5 mM), EYH_2 (15 µM), $CuBr_2$ (0.3 mM), Me_6TREN (0.45 mM), DMSO (10% v/v). Then 4.4 mL of the ATRP cocktail was added to a 1-dram (12/96 mm) vial equipped with a magnetic stir bar. The polymerization mixture in an uncapped vial was stirred at 500 rpm for 30–40 min under green LEDs (520 nm, 9.0 mW/cm^2). Samples were drawn at time intervals to monitor by ^{1}H NMR in D_2O and SEC techniques. Poly(HEA) and poly(MSEA) were analyzed by SEC (DMF as an eluent) calibrated by PMMA standards (Figure S5), while poly(CBA) was analyzed by SEC-MALS (1X DPBS as an eluent) (Figure S6).

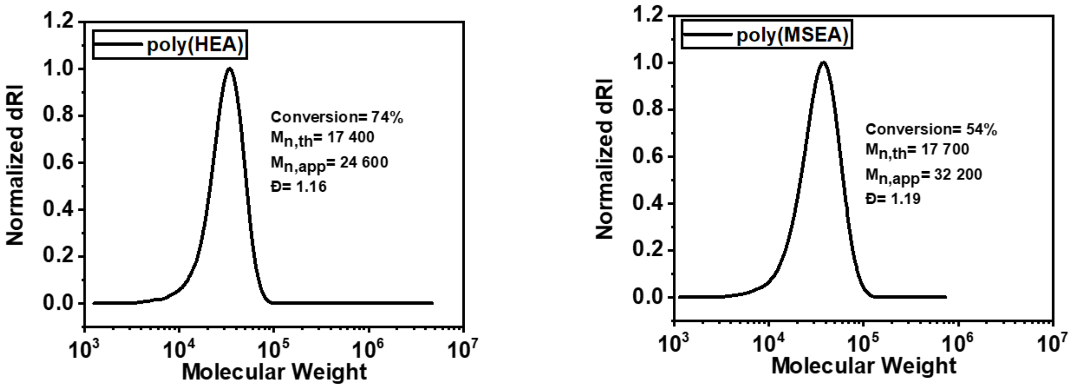

Figure S5. SEC traces of poly(HEA) and poly(MSEA).

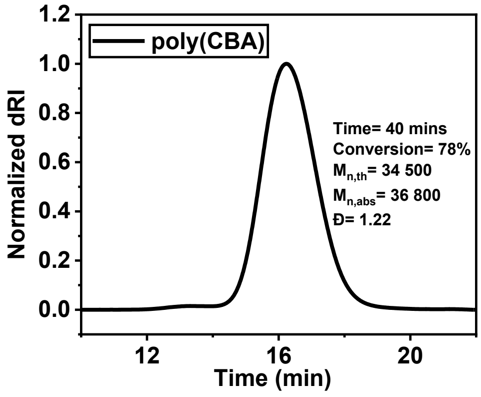

Figure S6. SEC-MALS traces of poly(CBA).

Synthesis of block copolymers by *in-situ* chain extension (Figure 5a)

Synthesis of linear poly($OEOA_{480}$) (DP_T = 100)

The ATRP cocktail (5 mL) with a target DP_T = 100 was prepared according to the general procedure. The final concentrations were: $OEOA_{480}$ (300 mM), HO-EBiB (3.0 mM), EYH_2 (15 µM), $CuBr_2$ (0.3 mM), Me_6TREN (0.9 mM), DMSO (10% v/v). Then 4.4 mL of the ATRP cocktail, ([$OEOA_{480}$]/[HO-EBiB]/[EYH_2]/[$CuBr_2$]/[Me_6TREN] = 100/1/0.05/0.1/0.15) was added to a 1-dram (12/96 mm) vial equipped with a magnetic stir bar. The polymerization mixture in an uncapped vial was stirred at 500 rpm for 40 min under green LEDs (525 nm, 25.0 mW/cm^2). The macroinitiator poly($OEOA_{480}$) was synthesized with 82% monomer conversion ($M_{n,app}$= 29 400, $Đ$ = 1.12).

In-situ chain extension of linear poly($OEOA_{480}$) (DP_T = 100)

A sample (1 mL) of linear poly ($OEOA_{480}$) (DP_T = 100) was then taken from the post-polymerization mixture and used without further purification to prepare an ATRP cocktail with additional $OEOA_{480}$ (DP_T = 500) as follows. In a 5 mL volumetric flask, 720 mg of $OEOA_{480}$ was weighed and mixed with a crude sample of poly($OEOA_{480}$) solution (1 mL). $CuBr_2$ stock (200 µL), Me_6TREN stock (100 µL), EYH_2 stock (50 µL) and 10X PBS solution (500 µL) were then added. 4.4 mL of the ATRP cocktail with a DP_T = 500 ($OEOA_{480}$) was added to a 1-dram (12/96 mm) vial equipped with a magnetic stir bar. The polymerization mixture in an uncapped vial was stirred at 500 rpm for 40 min under green LEDs (525 nm, 25.0 mW/cm^2). The sample was taken and analyzed by ^{1}H NMR and SEC techniques. SEC analysis showed a clear shift toward higher molecular weights without any shoulder or tailing at lower molecular weights (conv. = 61%, $M_{n,app}$ = 120 000, $Đ$ = 1.26), (Figure 4a).

The polymer macroinitiator poly($OEOA_{480}$) was also extended with other water-soluble acrylate monomers. In this case, in a 5 mL volumetric flask, 1.5 mmol of HEA or MSEA was weighed and mixed with a crude sample poly($OEOA_{480}$) solution (1 mL). $CuBr_2$ stock (200 µL), Me_6TREN stock (100 µL), EY stock (50 µL) and 10X PBS solution (500 µL). 4.4 mL of the ATRP cocktail with a target DP = 500 was added to a 1-dram (12/96 mm) vial equipped with a magnetic

stir bar. The polymerization mixture was stirred at 500 rpm for 30 min under green LEDs (525 nm, 25.0 mW/cm^2). The resultant copolymers were analyzed by ^{1}H NMR and SEC (Figure S7). The block copolymers of desired molecular weight could be prepared, although they showed a slightly broad distribution. Further optimizations may be needed to achieve better control over the block copolymers comprising of different monomers.

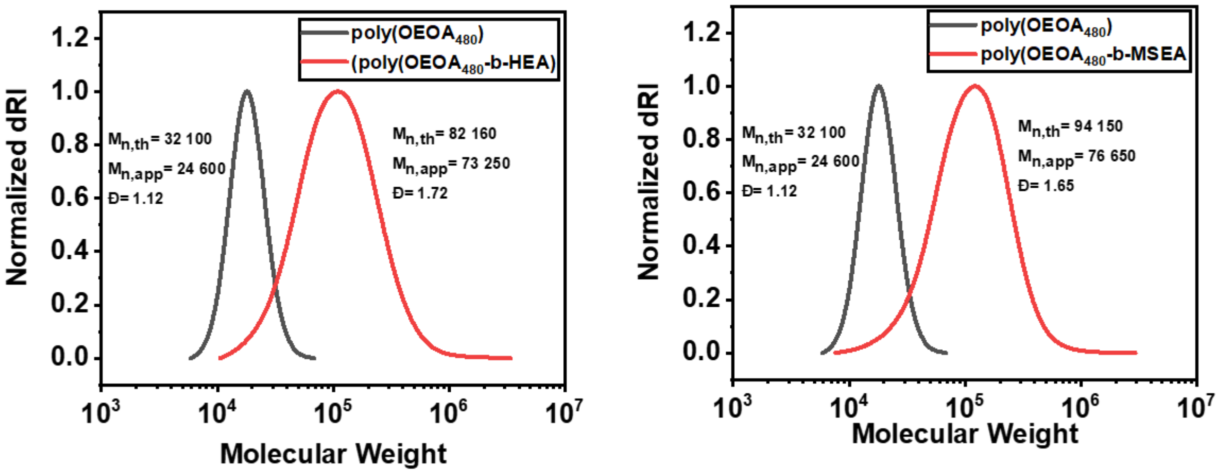

Figure S7. SEC traces for block copolymers of poly($OEOA_{480}$-*b*-HEA) and poly($OEOA_{480}$-*b*-MSEA).

Synthesis of protein-polymer hybrids

Synthesis and characterization of CT macroinitiators with 7 and 12 ATRP initiators

The synthesis of chymotrypsin macroinitiator was achieved by reaction of NHS-functionalized ATRP initiator (NHS-Br) with purified CT. NHS-Br (146 mg, 437 µmol) was dissolved in DMSO (0.5 mL), divided into three portions and added to CT (260 mg, 10.4 µmol) solution (20 mL, 100 mM sodium phosphate buffer (pH = 8.0)) every 30 minutes. After stirring the mixture in a refrigerator (4 °C) for 3 hours, the functionalized CT was purified by dialysis using a 15 KDa molecular weight cut-off dialysis tube against 25 mM sodium phosphate (pH = 8.0) and deionized water in a refrigerator for 24 hours. The final product was isolated by lyophilization. The number of functionalized sites on CT was determined by fluorescamine assay as reported in our previous publication.[2] Increased functionalization of CT was achieved by stopping the reaction at a longer time.

General procedure for the synthesis of CT-poly($OEOA_{480}$) biohybrids (Table 4)

The ATRP cocktail in a 2 mL volumetric flask was prepared according to the general procedure. The final concentrations were: $OEOA_{480}$ (100 mM), CT-7 or -12 (0.285-0.071 mM), EYH_2 (10 µM), $CuBr_2$ (0.2 mM), Me_6TREN (0.2 mM), DMSO (10% v/v) for polymerization at 100 mM monomer concentration, and $OEOA_{480}$ (300 mM), CT-12 (0.125-0.083 mM), EYH_2 (15 µM), $CuBr_2$ (0.3 mM), Me_6TREN (0.45 mM), DMSO (10% v/v) for polymerization at 300 mM monomer concentration. Then 2 mL of the ATRP cocktail was transferred to a 2 mL HPLC vial and placed in the Lumidox® photoreactor equipped with green light LEDs (527 nm, 125 mW/cm^2) and a cooling system. The polymerization was stopped after 3 h by turning the light off. For SEC analysis, the polymerization solution was purified by dialysis using a 15 KDa molecular weight cut-off dialysis tube in deionized water.

CT-7-poly($OEOA_{480}$) biohybrid enzymatic activity

Enzymatic activity of CT-7-poly($OEOA_{480}$) (Table 4, entry 6) was measured as an example. In a cuvette, 0.1 M sodium phosphate buffer (900−990 µL, pH = 8.0), substrate (*N*-Succinyl-L-Ala-L-Ala-L-Pro-L-Phe-p-nitroanilide, 0−90 µL, 6 mg/mL in DMSO), and CT-7-poly($OEOA_{480}$) (10 µL, 0.1 mg/mL) were mixed. The rate of hydrolysis was determined by recording the increase in absorbance at 412 nm for the first 45 s after mixing. K_M and k_{cat} values were calculated using Originlab software by fitting with the *Michaelis-Menten* function.

Table S3. Michaelis-Menten parameters of hydrolysis of *N*-Suc-AlaAlaProPhe-pNA for CT (native) and CT-7-poly($OEOA_{480}$).

Sample	V_{max} (µM s^{-1})	K_M (µM)	k_{cat} (s^{-1})	k_{cat}/K_M (µM^{-1} s^{-1})
CT-7-poly($OEOA_{480}$) (Table 4, entry 6)	0.40 ± 0.02	202.4 ± 26.2	9.9 ± 0.4	0.05 ± 0.01
CT (native)	1.37 ± 0.06	101.6 ± 17.4	34.3 ± 1.6	0.34 ± 0.06
